# Extracellular Matrix in Development and Disease

**DOI:** 10.3390/ijms20010205

**Published:** 2019-01-08

**Authors:** Julia Thom Oxford, Jonathon C. Reeck, Makenna J. Hardy

**Affiliations:** 1 Center of Biomedical Research Excellence in Matrix Biology, Boise State University, Boise, ID 83725-1511, USA; jonathonreeck@boisestate.edu (J.C.R.); makennahardy@u.boisestate.edu (M.J.H.); 2 Biomolecular Research Center, Boise State University, Boise, ID 83725-1511, USA; 3 Biomolecular Sciences Graduate Program, Boise State University, Boise, ID 83725, USA; 4 Department of Biological Science, Boise State University, Boise, ID 83725-1515, USA

**Keywords:** extracellular matrix, hereditary diseases, reproduction, cancer, muscle, tissue engineering, integrins

## 1. Introduction

The evolution of multicellular metazoan organisms was marked by the inclusion of an extracellular matrix (ECM), a multicomponent, proteinaceous network between cells that contributes to the spatial arrangement of cells and the resulting tissue organization. The development of an ECM that provides support in larger organisms may have represented an advantage in the face of selection pressure for the evolution of the ECM.

The study of the ECM, or material outside the cells of tissues, began with the cell theory of life in the 1850s [1]. Advances in our understanding of the composition of the ECM occurred prior to 1975 with the application of new techniques in chemical, physical, and biological research [2]. These new techniques provided information about the molecular structure of collagen and the chemical nature of the crosslinks that stabilize the collagen higher-ordered structure. Analysis of biosynthetic pathways has revealed that ECM components are synthesized and secreted by conserved mechanisms throughout metazoan organisms. Additionally, a comparison between organisms has revealed that ECM proteins are oligomeric, commonly forming trimers and pentamers of protein monomers that often possess repeated motifs that predate the evolutionary origin of animals. With the advance of molecular biology and cell biology fields, new collagens and noncollagenous constituents were discovered and characterized to give our current view of the ECM as a complex and dynamic biomaterial. Today, we recognize that the ECM plays an essential role in development and disease, supporting cell survival, differentiation, and tissue organization.

The ECM has been the focus of research for over a century. Scientists strive to understand the processes involved in biosynthesis and turnover, and many important discoveries have helped to pave the way to the application of extracellular matrices in ways that translate to patient care. Despite these efforts, there are still many unanswered questions that remain. This book deals with molecular and cellular aspects of the role of ECM in development and disease. Cells exist in three-dimensional scaffolding that holds together the millions of cells that make up our blood vessels, organs, skin, and all tissues of the body. The matrix serves as a reservoir of signaling molecules as well. In bacterial cultures, biofilms form as an ECM and play essential roles in disease and drug resistance. Topics such as matrix structure and function, cell attachment, and cell surface proteins mediating cell–matrix interactions, synthesis, regulation, composition, structure, assembly, remodeling, and function of the matrix are covered. A common thread uniting the topics is the essential nature that the matrix plays in normal development and pathophysiology. Providing new knowledge will lead us to improved diagnostics, preventions to disease progression, and therapeutic strategies for repair and regeneration of tissues.

## 2. Extracellular Matrix and Hereditary Diseases

The link between ECM molecules and phenotypic characteristics of specific hereditary diseases is reviewed by Arseni and colleagues [3], with an emphasis on the most prevalent of all ECM proteins, collagens, and mutations that result in bone fragility, muscle weakness, and skin defects. The authors review the dynamic state of collagen molecules (chains), domains, and molecular interaction, highlighting the dynamic nature of the ECM and the complexity of understanding how mutations contribute to hereditary diseases.

Extending the focus on collagens and point mutations that lead to inherited diseases, primarily affecting the cartilage and skeletal systems such as Stickler syndrome and congenital spondyloepiphyseal dysplasia, Chakkalakal and colleagues [4] reported new findings on mutations of collagen type II that impact protein secretion, the unfolded protein response, and ultimately, cell survival. This research reinforces the findings that collagen mutations near the C-terminus contribute to disease by causing retention in the ER and inducing the ER-stress response.

Another prevalent ECM molecule is hyaluronan. Stridh and colleagues [5] provided novel findings on the role of hyaluronan produced by renomedullary interstitial cells in kidney function. Hyaluronan is vital to the balance of fluid electrolytes. Through this knowledge, they discovered a rapid hyaluronan turnover mechanism alternative to regulating synthesis. They build on previous work showing that hyaluronan levels change corresponding to body hydration levels. This new work adds that hormone-regulated hyaluronidase activity decreases hyaluronan.

## 3. Extracellular Matrix and Reproduction

Hyaluronan plays an essential role during embryonic development. Eva Nagyova [6] reviews the biological role of hyaluronan-rich oocyte-cumulus ECM in female reproduction. In her review, she focuses on key molecules that play an important role in the formation of the cumulus ECM, generated by the oocyte-cumulus complex. One of these key molecules, inter-alpha-trypsin inhibitor protein forms covalent interactions with hyaluronan to create the major structural component of the cumulus ECM.

During implantation and placentation, plasminogen activator inhibitor type 1 (PAI-1) is responsible for inhibiting ECM degradation, thereby causing inhibition of trophoblast invasion. An increase of PAI-1 in the blood is associated with an increased risk for infertility and pregnancy complications. PAI-1 levels are increased in patients with recurrent pregnancy losses, preeclampsia, intrauterine growth restriction, gestational diabetes mellitus in the previous pregnancy, endometriosis and polycystic ovary syndrome. Ye and colleagues [7] provide an overview of the current knowledge of the role of PAI-1 in reproductive diseases. Based on this review, PAI-1 may represent a promising biomarker for reproductive diseases.

## 4. Extracellular Matrix and Cancer

Cancer is defined as a disease of uncontrolled cell proliferation and dysregulation of the microenvironment, which included the ECM. Walker et al. [8] reviewed the role of the ECM in cancer development and progression from a biochemical as well as biophysical perspective while Wang et al. [9] reviewed the link between increased risk of thrombosis in cancer patients. Also related to cancer is the recent article by Del Ben et al. [10], which focuses on the role of the ECM molecule vitronectin in nonalcoholic steatohepatitis (NASH), a leading cause of liver cirrhosis and hepatocellular carcinoma. The results of their studies indicate that a fragment of vitronectin may serve as an effective biomarker for noninvasive diagnosis of NASH. Rijal et al. [11] and Song et al. [12] address novel three-dimensional ECM scaffolds with application to cancer research. Rijal et al. [11] reported the generation of breast-specific ECM that can be used to form a hydrogel porous scaffold for studying human breast cancer. Song et al. [12] further reviewed the three-dimensional scaffolds that may accurately reflect the complexity of native tissues and cancer microenvironments for future studies.

## 5. Extracellular Matrix and Muscle

Mast cells have been implicated in the development of cardiac fibrosis. The controversial role that mast cells may play in promoting a profibrotic environment in cardiac muscle is reviewed by Levick and Widiapradja [13]. The role of the members of the thrombospondin family is reviewed by Chistiakov et al. [14], highlighting the potential for the therapeutic treatment of cardiovascular disease by targeting cardiac thrombospondin-mediated signaling in cardiovascular disease. Rohm and colleagues [15] present their current findings that will impact patients with pulmonary hypertension, right ventricular dysfunction and vascular remodeling. In their studies, they investigated the potential of elevated levels of a novel isoform of tenascin-C as a diagnostic serum biomarker.

McRae et al. [16] provide novel information about the dysregulated ECM in Duchenne muscular dystrophy and the effect of glucocorticoids on the versican-rich transitional matrix in both hindlimb and diaphragm muscle of a mouse model system. Versican may serve as a therapeutic target to promote myoblast fusion during muscle development and regeneration in patients with Duchenne muscular dystrophy.

Using a zebrafish model for muscle development, Dancevic et al. [17] demonstrated that ADAMTS5 plays an essential role in somite differentiation due to a new and novel role associated with the sonic hedgehog (shh) signaling pathway, in addition to its role in ECM remodeling. Their results suggest that a loss of ADAMTS5 causes changes in shh signaling which has been associated with musculoskeletal diseases.

## 6. Extracellular Matrix and Tissue Engineering Applications

Alvarèz Fallas et al. [18] and Uricuiolo and De Coppi [19] addressed the challenge of muscle regeneration. Alvarèz Fallas et al. [18] addressed the application of decellularized skeletal muscle tissue that preserves the tissue-specific ECM to support tissue engineering and regenerative medicine using both ex vivo and in vivo models. Their work shows that decellularized diaphragm is a suitable scaffold for skeletal muscle tissue engineering and regeneration. Uricuiolo and De Coppi [19] review the literature on the repair of volumetric muscle loss and the promising aspects of applying decellularized tissues as natural scaffolds for therapeutic outcomes.

## 7. Integrins

Finally, LaFoya and colleagues [20] review the many diverse roles for members of the integrin family. Integrins are traditionally thought of as receptors for the molecules of the ECM, however, in addition to this important role, they also play roles in cell–cell interactions, as growth factors and hormone receptors, and as interaction sites for viruses and bacteria. The many non-ECM integrin ligands are actively being characterized and may find many interesting uses in biotechnology. For example, it has been shown that RGD peptides attached to liposomes or viral particles increase tissue specificity.

## 8. Concluding Remarks

We thank all the authors who have generously contributed their articles to this book. By disseminating and sharing our research, we ensure that advances in the field can be made. It is by dissemination of our work that the next innovation will arise.

## Abbreviations

ECM	Extracellular matrix
ER	Endoplasmic reticulum
PAI-1	Plasminogen activator inhibitor type 1
NASH	nonalcoholic steatohepatitis
ADAMTS5	A disintegrin and metalloproteinase with thrombospondin motifs 5
Shh	Sonic hedgehog
RGD	Arginine-glycine-aspartic acid
